# Age-related changes to the attentional modulation of temporal binding

**DOI:** 10.3758/s13414-023-02756-8

**Published:** 2023-07-26

**Authors:** Jessica L. Pepper, Barrie Usherwood, Theodoros M. Bampouras, Helen E. Nuttall

**Affiliations:** 1 Department of Psychology, Fylde College, Lancaster University, Lancaster, UK LA1 4YF; 2 School of Sport and Exercise Sciences, Liverpool John Moores University, Liverpool, UK L3 3AF

**Keywords:** Ageing, Attention, Temporal binding, Multisensory integration

## Abstract

During multisensory integration, the time range within which visual and auditory information can be perceived as synchronous and bound together is known as the temporal binding window (TBW). With increasing age, the TBW becomes wider, such that older adults erroneously, and often dangerously, integrate sensory inputs that are asynchronous. Recent research suggests that attentional cues can narrow the width of the TBW in younger adults, sharpening temporal perception and increasing the accuracy of integration. However, due to their age-related declines in attentional control, it is not yet known whether older adults can deploy attentional resources to narrow the TBW in the same way as younger adults. This study investigated the age-related changes to the attentional modulation of the TBW. Thirty younger and 30 older adults completed a cued-spatial-attention version of the stream-bounce illusion, assessing the extent to which the visual and auditory stimuli were integrated when presented at three different stimulus-onset asynchronies, and when attending to a validly cued or invalidly cued location. A 2 × 2 × 3 mixed ANOVA revealed that when participants attended to the validly cued location (i.e., when attention was present), susceptibility to the stream-bounce illusion decreased. However, crucially, this attentional manipulation significantly affected audiovisual integration in younger adults, but not in older adults. These findings suggest that older adults have multisensory integration-related attentional deficits. Directions for future research and practical applications surrounding treatments to improve the safety of older adults’ perception and navigation through the environment are discussed.

## Introduction

During multisensory processing, a key factor required to ascertain whether two sensory inputs are related is their temporal proximity (Hillock et al., 2011; Vroomen & Keetels, 2010). If auditory and visual inputs are presented closely together in time, they are more likely to be perceived as originating from the same event (Stevenson et al., 2012; Meredith & Stein, 1986) and bound together into a single, multisensory perceptual entity (Spence & Squire, 2003; Zampini et al., 2005). The adjustable time range within which visual and auditory stimuli can be perceived as synchronous and thus have an increased likelihood of being integrated is known as the temporal binding window (TBW; Bedard & Barnett-Cowan, 2016; Mégevand et al., 2013; Mozolic et al., 2012). The TBW allows two congruent sensory inputs to be integrated even if there is a degree of temporal discrepancy (e.g., due to differences in the speed of light versus sound, or differences in sensory propagation time; Mégevand et al., 2013; Pöppel et al., 1990; Stevenson et al., 2012). Likewise, bimodal sensory information that does not occur within the limits of the TBW will not be perceived concurrently, and therefore will not be bound together and can correctly remain discrete (Stevenson et al., 2012).

The width of the TBW is believed to widen with healthy ageing (Bedard & Barnett-Cowan, 2016; Diederich et al., 2008; Poliakoff et al., 2006; Setti et al., 2014). It has been well established in psychophysical research that older adults integrate more sensory information than younger adults, showing faster reaction times and greater accuracy in response to multisensory stimuli than unisensory stimuli (Laurienti et al., 2004, 2006; Peiffer et al., 2007). Recent research has postulated that this multisensory ‘enhancement’ exhibited by older adults may be due to a combination of their wider TBW and their attentional deficits. Specifically, age-related deficits in allocating the necessary attentional resources required for the top-down modulation of sensory processing could mean that, for older adults, the boundaries of the TBW are less restricted (Setti et al., 2011). As such, due to having a greater time range over which integration can occur, older adults then demonstrate increased integration across multiple modalities (Brooks et al., 2018) compared with the integration exhibited by younger adults (Laurienti et al., 2006; Peiffer et al., 2007). This increased integration is advantageous for older adults when the unisensory inputs are congruent and should contextually be bound together (Laurienti et al., 2006) yet can cause errors in perceptual performance if incongruent information is integrated when it should remain discrete (Poliakoff et al., 2006; Setti et al., 2014).

In everyday life, incorrectly identifying whether stimuli from different modalities should be integrated or segregated can lead to inaccurate and dangerous perceptions of the immediate environment (Bedard & Barnett-Cowan, 2016; Wise & Barnett-Cowan, 2018). This is evident in the fact that wider TBWs are associated with an increased risk of falls in older adults (Mahoney et al., 2014, 2019; Peterka, 2002; Setti et al., 2011)—when task-irrelevant sensory information is incorporated into the representation of the physical world, this could provoke distractibility and lead to a fall (Peiffer et al., 2007; Setti et al., 2011). From this safety perspective, it is clear how important it is to investigate if and how the TBW can be narrowed by attentional control, in order to sharpen perception and increase the ability of older adults to keep irrelevant information separate from meaningful sensory inputs in their dynamic environment.

Ostensibly, manipulating attentional cues could be a promising mechanism to narrow the TBW of older adults (Setti et al., 2011). However, the limited evidence surrounding how attentional abilities change with healthy ageing suggests that older adults find it more difficult than younger adults to focus their attention on only task-relevant information and inhibit the processing of task-irrelevant information (Gazzaley et al., 2005; Healey et al., 2008; Park & Reuter-Lorenz, 2009; Zhuravleva et al., 2014)—this has been termed the ‘inhibitory deficit hypothesis’ (Alain & Woods, 1999; Hasher & Zacks, 1988).

**Fig. 1:**
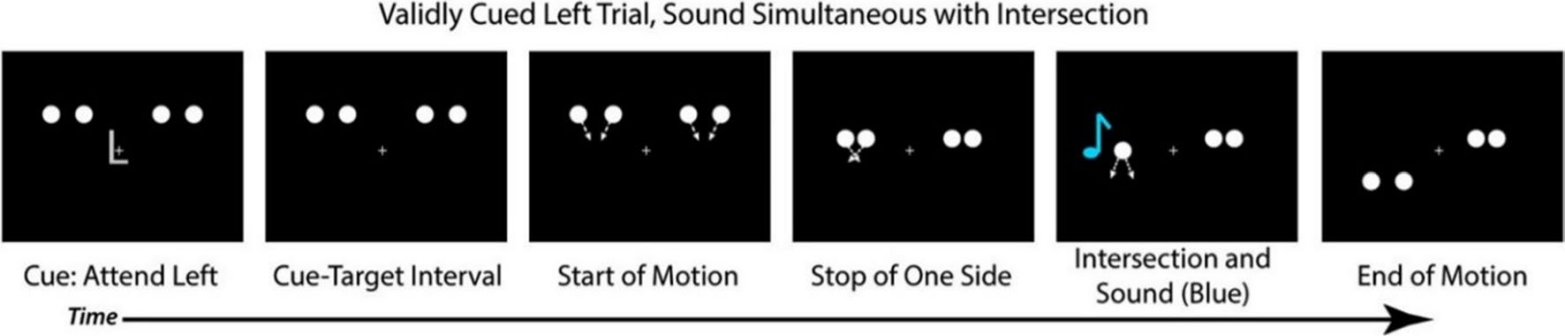
Diagram of the cued-spatial-attention stream-bounce illusion. Image taken from the published manuscript of Donohue et al. (2015). (Colour figure online)

Donohue et al. (2015) implemented a cued-spatial-attention version of the stream-bounce illusion with younger adults to investigate how attentional mechanisms modulate the width of the TBW. In the stream-bounce illusion, the visual motion of the circles is always identical and task-relevant; however, when a task-irrelevant sound is played at the same time as the circles intersect, the auditory and visual sensory inputs are bound together (Fig. 1). This results in the perception that the circles “bounced off” rather than “passed through” each other. Donohue et al.’s findings indicated that attending to the validly cued location (i.e., viewing the full visual motion of the circles) could narrow the width of the TBW in younger adults, producing more accurate judgements regarding the temporal alignment of the visual and auditory information, and thus whether they should be integrated.

Despite this, it is not yet known whether older adults are able to deploy the necessary attentional resources required to narrow their TBW as effectively as younger adults can. The present study investigated whether there are age-related changes in this attentional modulation of the TBW, comparing the judgements and reaction times of younger and older adults in a cued-spatial-attention version of the stream-bounce task.

Firstly, it is predicted that due to their wider TBW, older adults will be more prone to binding together the visual input of the circles intersecting with the auditory input of the task-irrelevant tone, even if they do not occur synchronously. This will manifest as older adults providing a greater proportion of “bounce” responses in the stream-bounce illusion than younger adults at longer stimulus-onset asynchronies (SOAs), confirming previous research (Bedard & Barnett-Cowan, 2016).

Secondly, it is predicted that across age groups, the proportion of “bounce” responses will be greater in the invalidly cued conditions than in the validly cued conditions. Participants are likely to display increased uncertainty if they are not attending to the full “X” shaped motion of the visual stimuli, the TBW will not be narrowed due to the absence of attention, and participants will perceive the visual and auditory information as synchronous at longer SOAs (Donohue et al., 2015).

Finally, it is predicted that due to the postulated attentional deficits of older adults, they will display less of a difference in the proportion of “bounce” responses in the validly cued versus invalidly cued conditions, compared with the difference produced by younger adults. In other words, the attentional manipulation may have less of an effect on multisensory integration in older adults than in younger adults. These a priori predictions were preregistered prior to data collection on www.aspredicted.org, project ID #65513 (https://aspredicted.org/zx9ev.pdf).

## Method

### Participants

This study used a total of 60 participants; 30 younger adults (15 males, 15 females) between 18 and 35 years old (*M* = 21.37, *SD* = 1.30) and 30 older adults (11 males, 19 females) between 60 and 80 years old (*M* = 67.91, *SD* = 4.71). This sample size was determined via an a priori power analysis using the ANOVA_exact Shiny app (Lakens & Caldwell, 2019; see preregistration on www.aspredicted.org, project ID #65513, https://aspredicted.org/zx9ev.pdf). Based on the large effect size (Cohen’s *f* = 0.4) from similar studies (Basharat et al., 2019; Bedard & Barnett-Cowan, 2016; Chen et al., 2021; Donohue et al., 2015), an alpha value of *p* = .05 and power of 80%, the minimum sample size required was 30 participants per group.

All participants were fluent English speakers. Participants were required to have normal or corrected-to-normal vision, screened for via self-report. Participants were ineligible to proceed with the experiment if they had a history or current diagnosis of neurological conditions (e.g., epilepsy, mild cognitive impairment, dementia, Parkinson’s disease) or learning impairments (e.g., dyslexia), or had hearing loss resulting in the wearing of hearing aids.

Participants were recruited via opportunity sampling; the majority of younger participants were students at Lancaster University and were known to the researcher, whilst the majority of older participants were members of the Centre for Ageing Research at Lancaster University. All participants provided informed consent.

### Prescreening tools

Participants were asked to complete two prescreening questionnaires using Qualtrics survey software (www.qualtrics.com), to assess their eligibility for the study.

#### Speech, Spatial and Quality of Hearing Questionnaire (SSQ; Gatehouse & Noble, 2004)

Participants rated their hearing ability in different acoustic scenarios using a sliding scale from 0 to 10 (0 = *not at all*; 10 = *perfectly*). Whilst, at present, no defined cut-off score on the SSQ is available as a parameter to inform decision-making, previous studies have indicated that a mean score of 5.5 is indicative of moderate hearing loss (Gatehouse & Noble, 2004). As a result, people whose average score on the SSQ was lower than 5.5 were not eligible to participate in the experiment.

#### Informant Questionnaire on Cognitive Decline in the Elderly (IQ-CODE; Jorm, 2004)

Participants used a self-reported version of the IQ-CODE to rate how their performance in certain tasks now has changed compared with 10 years ago, answering on a 5-point Likert scale (1 = *much improved*; 5 = *much worse*). An average score of approximately 3.3 is the usual cut-off point when evaluating cognitive impairment and dementia (Jorm, 2004); therefore, people whose average score was higher than 3.3 were not eligible to participate in the experiment.

**Fig. 2:**
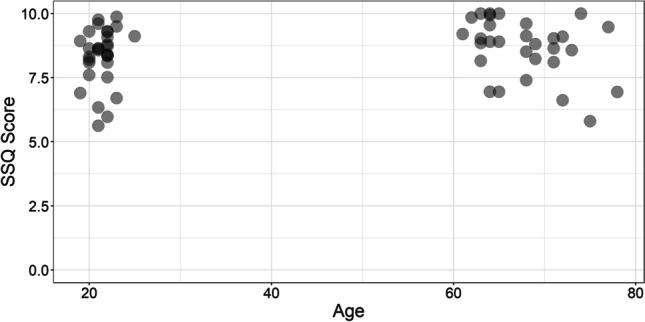
SSQ scores of younger and older adults. Each point represents the score of each individual participant

**Fig. 3:**
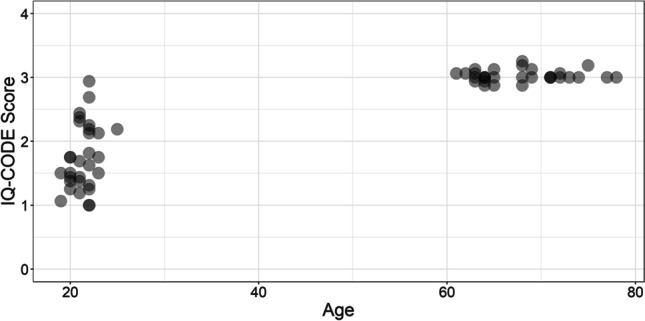
IQ-CODE scores of younger and older adults. Each point represents the score of each individual participant

The mean scores produced by younger and older adults in each prescreening questionnaire are displayed in Table 1, with individual scores displayed in Figs. 2 and 3. A Mann–Whitney *U* test revealed that there was no significant difference between age groups on the SSQ questionnaire [*U*(*N*_Younger_ = 30, *N*_Older_ = 30) = 353.00, *p* = .15]; however, there was a significant difference between age groups on the IQ-CODE questionnaire [*U*(*N*_Younger_ = 30, *N*_Older_ = 30) = 4.00, *p* < .001].

**Table 1:** Mean scores on the SSQ and IQ-CODE prescreening questionnaires, for both younger and older adults (standard deviations displayed in parentheses)

Age group	SSQ	IQ-CODE
Younger	8.34 *(1.10)*	1.74 *(0.51)*
Older	8.67 *(1.13)*	3.03 *(0.09)*

### Experimental design

This research implemented a 2 (age: younger vs older) × 2 (cue: valid vs invalid) × 4 (stimulus onset asynchrony [SOA]: visual only [VO] vs 0 milliseconds vs 150 milliseconds vs 300 milliseconds) mixed design, with age as a between-subjects factor and cue and SOA as within-subjects factors.

The experiment consisted of 16 different trial conditions (Table 2), randomized across all participants. Replicating the paradigm used by Donohue et al. (2015), the experimental block contained 72 validly cued trials and 24 invalidly cued trials, which were equally distributed between each side of the screen (left/right) and SOA conditions; this means that each participant completed 144 valid trials and 48 invalid trials for each SOA.

**Table 2:** Number of trials within each cue and SOA condition.

SOA (ms)	Cue			
	Valid (Left) *N*	Valid (Right) *N*	Invalid (Left) *N*	Invalid (Right) *N*
0	72	72	24	24
150	72	72	24	24
300	72	72	24	24
VO	72	72	24	24

### Stimuli and materials

Participants were asked to complete the experiment online, in a quiet room on a desktop or laptop computer with a standard keyboard. All participants were asked to wear headphones/earphones. A volume check was conducted at the beginning of the experiment; participants were presented with a constant tone and asked to adjust the volume of this tone to a clear and comfortable level.

The stimuli used in the task were replicated from Donohue et al. (2015). Due to the fact that the experiment was completed remotely on participants’ personal computers, we were unable to confirm whether the specifications of each monitor were identical. However, data recorded in Pavlovia confirmed that each participant experienced a refresh rate of 60 Hz. Each trial started with an attentional cue in the centre of the screen—a letter “L” or a letter “R” instructing participants to focus on the left or the right side of the screen. In addition to this, two pairs of circles were positioned at the top of the screen—one pair in the left hemifield and one pair in the right hemifield. Each circle was 1.5° in diameter and were presented 4° above the attentional cue; inner disks were 4.9° and outer disks were 10° left and right of the attentional cue. The attentional cue lasted for 1 second, and 650 milliseconds after this cue disappeared, the circles in each pair started to move towards each other downwards diagonally (i.e., the two left circles moving towards each other and the two right circles moving towards each other).

In the trials, one pair of circles moved towards each other, intersected, and continued on the same trajectory (fully overlapping and moving away from each other). This full motion of the circles formed an “X” shape, with the circles appearing to “stream” or “pass through” each other. On the opposite side of the screen, the other pair of circles stopped moving before they intersected, forming half of this “X” motion. On 75% of the trials, the full “X”-shaped motion appeared on the side of the screen that the cue directed participants towards (validly cued trials); on the other 25% of trials, the full motion occurred on opposite side of the screen to where the cue indicated, and the stopped motion occurred at the cued location (invalidly cued trials).

**Fig. 4:**
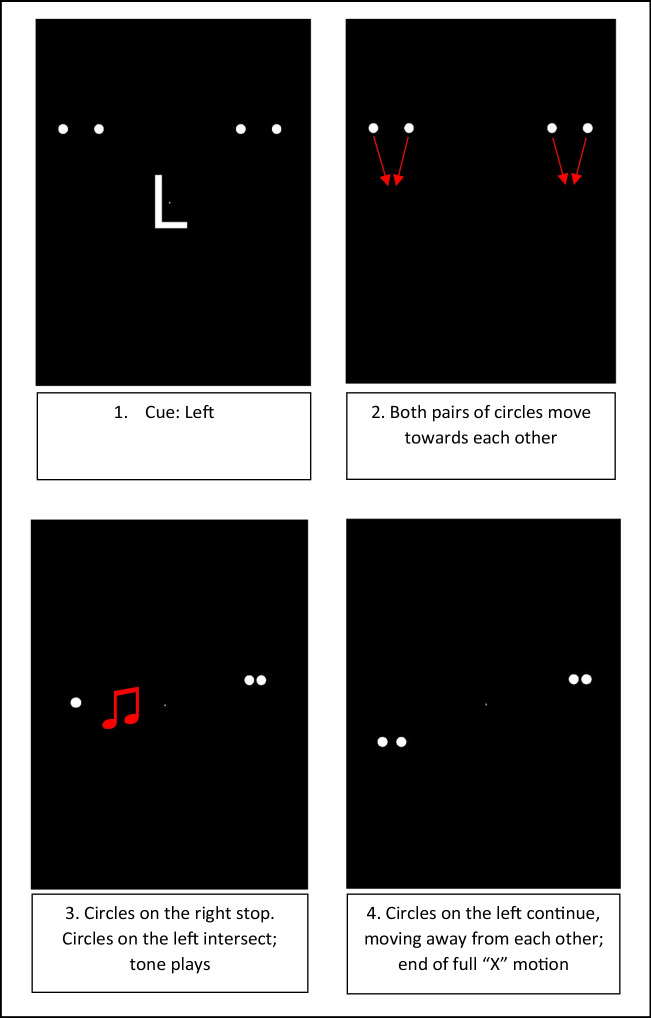
Screen captures of a validly cued trial (valid left), with an SOA of 0 ms (sound synchronous with intersection). Participants provided their pass/bounce judgement at the end of the trial. (Colour figure online)

In addition to these visual stimuli, on 75% of the trials, an auditory stimulus was played binaurally (500 Hz, 17 milliseconds), either at the same time as the circles intersected (0-ms delay), 150 ms after the intersection or 300 ms after the intersection. The remaining 25% of the trials were visual-only (i.e., no sound was played). Participants were told that regardless of whether a sound was played, they must make their pass/bounce judgements based on the full motion of the circles (the “X” shape), even if the full motion occurred at the opposite side of the screen that they were attending to. Screen captures of a validly cued, 0ms SOA trial are displayed in Fig. 4. Participation lasted approximately 1 hour. The experiment was built in PsychoPy2 (Peirce et al., 2019) and hosted by Pavlovia (www.pavlovia.org).

### Procedure

Prior to the experiment, a brief online meeting was organized between the participant and the researcher to explain the task and answer any questions. Participants were emailed a link to a Qualtrics survey, which included the participant information sheet, consent form, demographic questions and pre-screening questionnaires. If the participant was deemed eligible to take part in the experiment, Qualtrics redirected participants to the experiment in Pavlovia.

Participants were then presented with instructions detailing the attentional cue elements of the task and asking them to base their judgements on the full X-shaped motion of the stimuli. Participants were asked to press “M” on the keyboard if they perceived the circles to “pass through” each other or press “Z” if they perceived the circles to “bounce off” each other, answering as quickly and as accurately as possible.

Participants completed a practice block of 10 trials, then the test session commenced. After each set of 10 random trials, participants had the opportunity to take a break. Participants were provided with a full debrief upon completion of the experiment, and all participants could enter a prize draw to win one of two £50 Amazon vouchers.

### Statistical analyses

This study required four mixed ANOVAs—one for reaction times in visual-only unisensory conditions; one for reaction times in audiovisual multisensory (0 ms, 150 ms, 300 ms) conditions; one for bounce/pass judgements in visual-only unisensory conditions, and one for bounce/pass judgements in audiovisual multisensory (0 ms, 150 ms, 300 ms) conditions, following the analyses of Donohue et al. (2015).

#### Reaction times

For the first dependent variable of reaction time (RT), mean RTs were calculated for each participant in each Cue × SOA condition, representing the time taken, in milliseconds, for each participant to press “M” (“Pass Through”) or “Z” (“Bounce Off”) on the keyboard at the end of each trial. Responses (judgements or RTs) that were outside ±3 standard deviations were considered to be the result of different processes to the ones being examined (e.g., fast guesses or lack of attention; Whelan, 2008). Therefore, they were removed from subsequent analysis; this exclusion method was based on recommendations by Berger and Kiefer (2021). The RTs were then pooled and a grand mean was calculated and used for further analysis. As RTs are known to frequently deviate from normality (Whelan, 2008), the grand means were converted into *z*-scores, following the procedures recommended by Caldwell et al. (2019). A 2 (age: younger vs older) × 2 (cue: valid vs invalid) mixed ANOVA was then conducted on the* z*-score reaction times produced in the unisensory visual-only conditions, and a 2 (age: younger vs older) × 2 (cue: valid vs invalid) × 3 (SOA: 0 ms × 150 ms × 300 ms) mixed ANOVA was conducted on the *z*-score reaction times produced in the audiovisual multisensory conditions. As the unstandardized RT data showed a skewed distribution, medians, and IQRs are also displayed graphically using boxplots, as suggested by Whelan (2008).

#### Bounce/pass judgements

For the second dependent variable of the bounce/pass judgements, the percentage of “bounce” responses provided in each Cue × SOA condition was calculated for each participant. Firstly, to address the violation of ANOVA assumptions present with percentage data, the proportion of “bounce” responses produced in the unisensory visual-only conditions was converted into *z*-scores. A 2 (age: younger vs older) × 2 (cue: valid vs invalid) mixed ANOVA was conducted on these standardized data from the unisensory condition. In addition, the proportion of “bounce” responses produced in the audiovisual conditions (SOAs of 0 ms, 150 ms, and 300 ms) were pooled and a grand mean was calculated and used for further analysis. These grand means were converted into z-scores, following the procedures recommended by Caldwell et al. (2019). A 2 (age: younger vs older) × 2 (cue: valid vs invalid) × 3 (SOA: 0 ms vs 150 ms vs 300 ms) mixed ANOVA was then conducted on these standardized *z-*score data from the multisensory conditions. Post hoc paired-samples *t* tests were also used to investigate significant differences between the 0 ms, 150 ms, 300 ms and visual-only SOA conditions. Mauchly’s test of sphericity was violated for the main effect of SOA, therefore Greenhouse–Geisser adjusted *p*-values were used where appropriate.

After the 2 × 2 × 3 mixed ANOVA on the audiovisual data, to analyze pairwise comparisons in the significant interaction of age and cue, responses in each SOA condition were collapsed—that is, a grand mean percentage of “bounce” responses was calculated by averaging the percentage of “bounce” responses in the 0 ms, 150 ms, and 300 ms trials in the valid condition and in the invalid condition. This produced an overall valid and an overall invalid mean percentage of “bounce” responses for each participant. As with the reaction-time data and full bounce/pass data, these percentages were then pooled to allow calculation of the grand mean and subsequently converted to standardized *z*-scores, following the procedures recommended by Caldwell et al. (2019). Two separate one-way ANOVAs were conducted on this collapsed *z*-score data (“age” as the between-subjects factor, and valid or invalid as the within-subjects factor) to investigate differences between younger and older adults in the valid condition, and differences between younger and older adults in the invalid condition (Laerd, 2015). The datafile was then split by age, and a repeated-measures ANOVA using cue as the independent variable was conducted on this collapsed *z*-score data, to investigate differences between the proportion of “bounce” responses in the valid and invalid condition for younger adults, and in the valid and invalid condition for older adults (Laerd, 2015).

Data are presented as means and standard errors, and 95% confidence intervals are reported alongside the mean and the standard error for the bounce/pass analyses. Where two levels of a factor have been compared, the mean difference and standard error of this comparison has also been reported. An alpha level of .05 was used for all statistical tests. Statistical analyses were conducted using IBM SPSS Statistics for Windows (Version 25; IBM Corp., Armonk, NY, USA).

#### Deviations from preregistration

The analyses described in this manuscript differ from those outlined in the preregistration available on aspredicted.com. This is due to the implementation of recommendations from expert peer reviewers, which improved upon our original statistical analysis plan and validity of approach.

## Results

### Analysis of reaction-time (RT) data: Assessing the effectiveness of the attentional manipulation

**Fig. 5:**
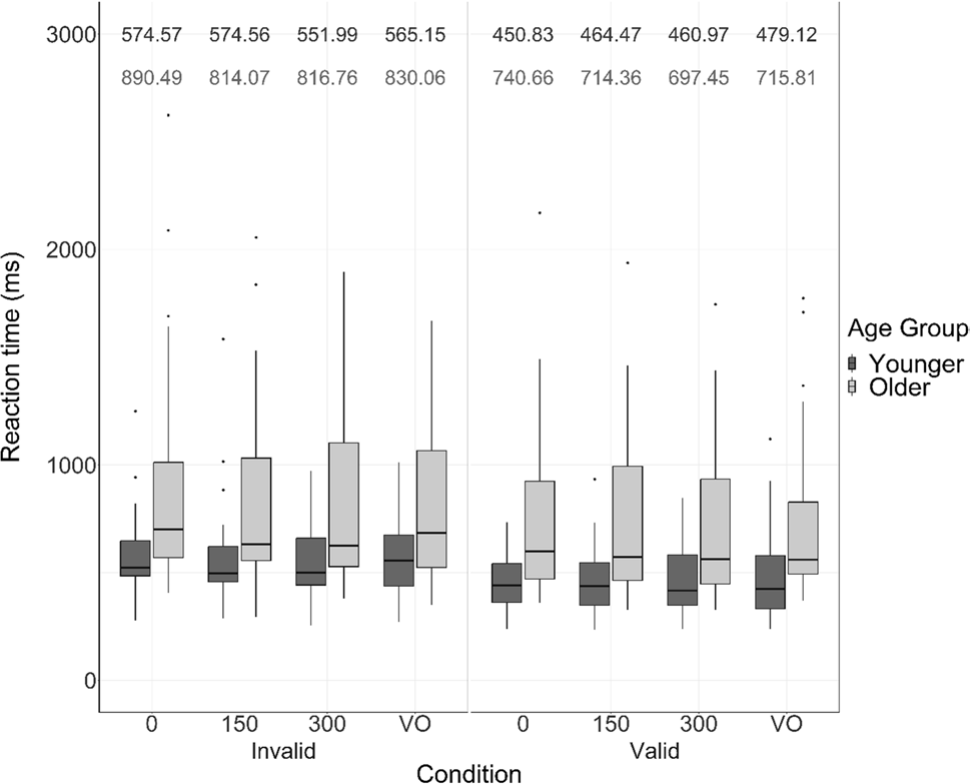
Participant reaction times (RTs), in milliseconds, in each SOA and cue condition. Black bars represent the RTs of younger adults; grey bars represent the RTs of older adults. Each bar displays the median, the lower quartile and the upper quartile for each condition (outliers plotted separately). Numbers at the top of each panel indicate mean RTs—younger adult RTs are presented in the upper row in black, and older adult RTs are presented in the lower row in grey

**Fig. 6:**
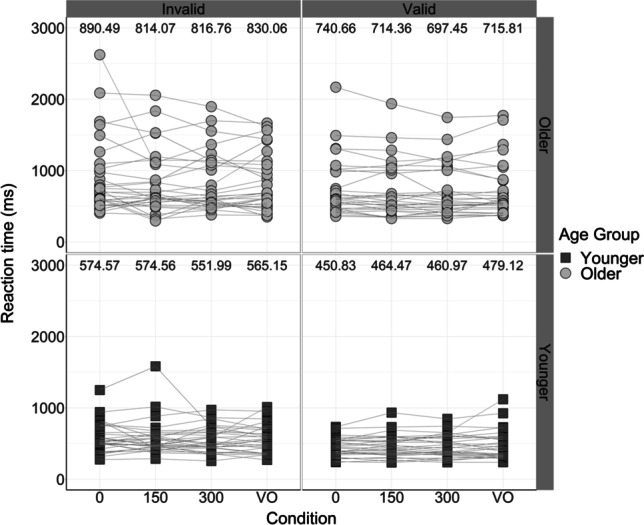
Participant reaction times (RTs), in milliseconds, in each SOA and cue condition. Black squares represent the RTs of younger adults; grey circles represent the RTs of older adults. Participants’ RTs across conditions are linked using lines. Numbers at the top of each panel display mean RTs in each condition

RTs in response to all trials (i.e., both “pass through” and “bounce” responses) were included in the analyses, as unlike other two-alternative forced choice tasks, there was no specific “correct” response. The mean RTs in each condition, for each age group, are displayed in Figs. 5 and 6.

It was important to compare RTs for valid trials, where the full “X” motion occurred at the cued side of the screen, with RTs for invalid trials, where the full “X” motion occurred at the opposite, uncued side of the screen, to ensure that participants abided by the attentional manipulation; validly cued trials should produce faster RTs than invalidly cued trials (Donohue et al., 2015). As a result, cue was the variable of interest in these RT analyses.

#### Reaction times: Unisensory conditions

A 2 (age: younger vs older) × 2 (cue: valid vs invalid) mixed ANOVA was conducted on the unisensory visual-only control conditions; there was a significant main effect of cue on the speed of key-press responses, *F*(1, 58) = 17.24, *p* < .001, η_p_^2^ = 0.23. Overall, participants were 100.14 ms faster at responding to validly cued trials compared with invalidly cued trials. In real-world contexts, simply attending to a specific location or modality speeds up reaction times, which is highly important for the safe and accurate perception of our environment (Mozolic et al., 2008). There was also a significant main effect of age on the speed of key-press responses, *F*(1, 58) = 10.98, *p* = .002, η_p_^2^ = 0.16—younger adults were 250.80 ms faster at responding than older adults. There was no significant interaction between age and cue, *F*(1, 58) = 0.34, *p* = .561, η_p_^2^ = 0.006.

#### Reaction times: Multisensory conditions

A 2 (age: younger vs older) × 2 (cue: valid vs invalid) × 3 (SOA: 0 ms vs 150 ms vs 300 ms) mixed ANOVA was then conducted on the RTs produced in the multisensory audiovisual conditions. These analyses indicated there was a significant main effect of cue on the speed of key-press responses, *F*(1, 58) = 25.44, *p* < .001, η_p_^2^ = 0.31—overall, participants were 115.53 ms faster at responding to the validly cued trials (*M* = 588.21 ms, *SE* = 36.76) compared with the invalidly cued trials (*M* = 703.74 ms, *SE* = 43.20), as displayed in Figs. 5 and 6. This suggests that the participants did attend to the validly cued side of the screen when directed, indicating that the attentional manipulation was effective. Using the same behavioural task, Donohue et al. (2015) found that their participants—a younger adult sample only—were 76 ms faster in the validly cued condition compared with the invalidly cued condition. As a result, the reaction time difference produced between cue conditions in the current experiment is meaningful and expected, yet larger than that produced in Donohue et al. (2015) due to the slower reaction times of older adults increasing the overall mean.

There was also a significant main effect of age on RTs, *F*(1, 58) = 11.98, *p* = .001, η_p_^2^ = 0.17—overall, younger adults (*M* = 512.90 ms, *SE* = 54.35) responded 226.07 ms faster than older adults (*M* = 778.97 ms, *SE* = 54.35), as displayed in Figs. 5 and 6. In a spatial attention task using younger and older adults, Madden (1990) found that younger adults were 184 ms faster than older adults, therefore it is fair to suggest that the reaction time difference generated by each age group in the current study is in line with previous literature. Whilst it was predicted that older adults would produce a slower response than younger adults, this result is indeed relevant to everyday life in that older adults could be slower at processing and responding to hazards in their dynamic environment. The resulting dangerous and inaccurate perception and action of older adults due to their slower reaction times may be associated with their increased risk of falls (Lajoie & Gallagher, 2004).

There was no significant main effect of SOA on RTs, *F*(2, 116) = 2.11, *p* = .126. There were no significant interactions between SOA and age, *F*(2, 116) = 1.98, *p* = .143, between SOA and cue, *F*(2, 116) = 0.710, *p* = .494, or between age and cue, *F*(1, 58) = 0.102, *p* = .750. Finally, the three-way interaction between cue, SOA and age was not significant, *F*(2, 116) = 0.249,* p* = .780.

### Analysis of bounce/pass judgements: Assessing the magnitude of multisensory integration

**Fig. 7:**
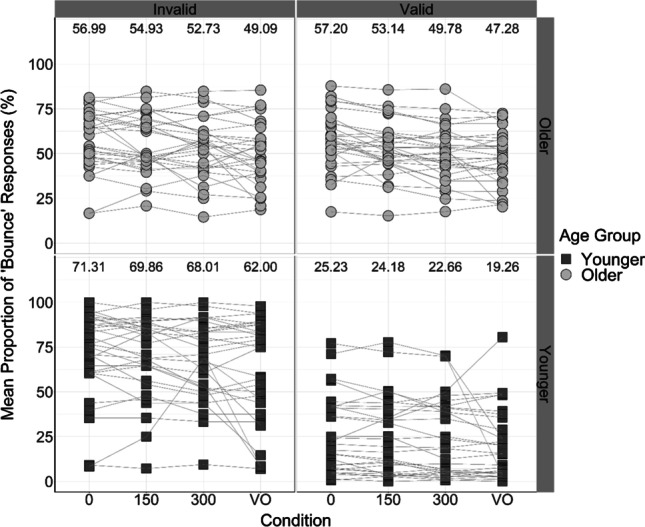
Mean proportion of “bounce” responses in each Cue × SOA condition for each participant. Black squares represent data of younger adults; grey circles represent the data of older adults. Participants’ “bounce” responses are linked across conditions using lines. Numbers at the top of each panel display the mean proportion of “bounce” responses in each condition

The purpose of analyzing the proportion of “bounce” responses in each condition was to assess the magnitude of multisensory integration across the different SOAs and across attentional cues. “Bounce” was the response of interest as it was indicative of the participant integrating the visual (circles intersecting) and auditory (tone playing) information in the trial. The percentage of “bounce” responses produced in each Cue × SOA condition was calculated for each participant. The mean proportion of “bounce” responses within each condition, for each age group, are displayed in Fig. 7.

**Fig. 8:**
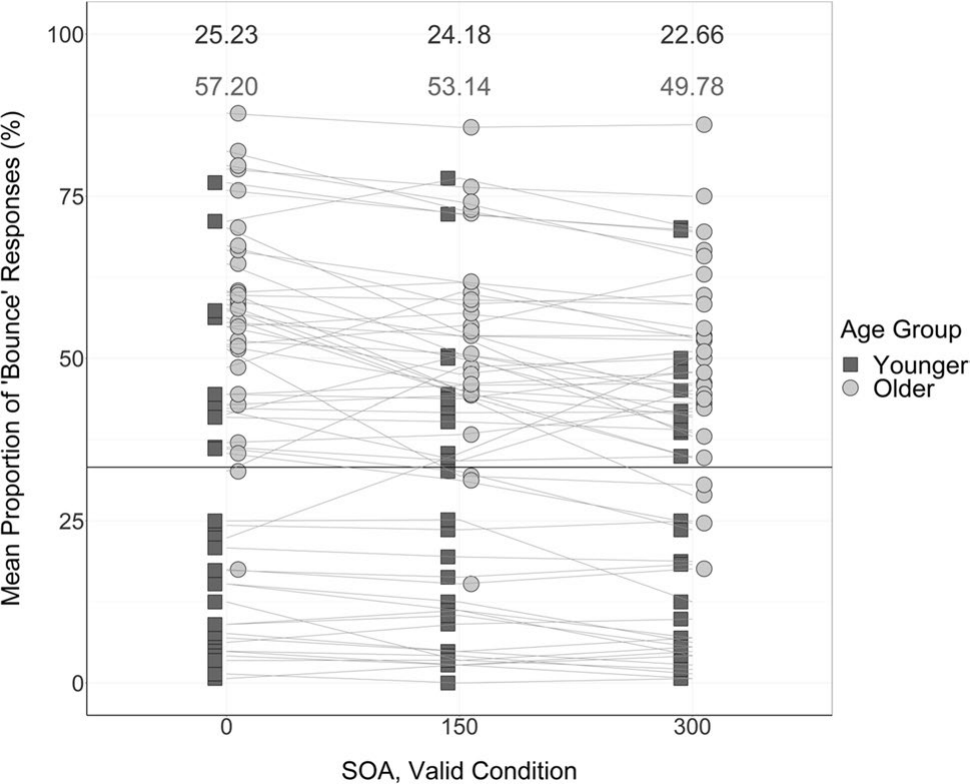
Mean proportion of “bounce” responses produced by each participant in each of the validly cued audiovisual conditions. Solid black horizontal reference line at 33.27% represents the mean proportion of “bounce” responses produced in the validly cued visual-only conditions. Black squares represent the data of younger adults; grey circles represent the data of older adults. Participant “bounce” responses are linked across conditions using lines. Numbers at the top of the figure display mean proportions of “bounce” responses in each condition—the means of younger adults are presented in the upper row in black; the means of older adults are presented in the lower row in grey

**Fig. 9:**
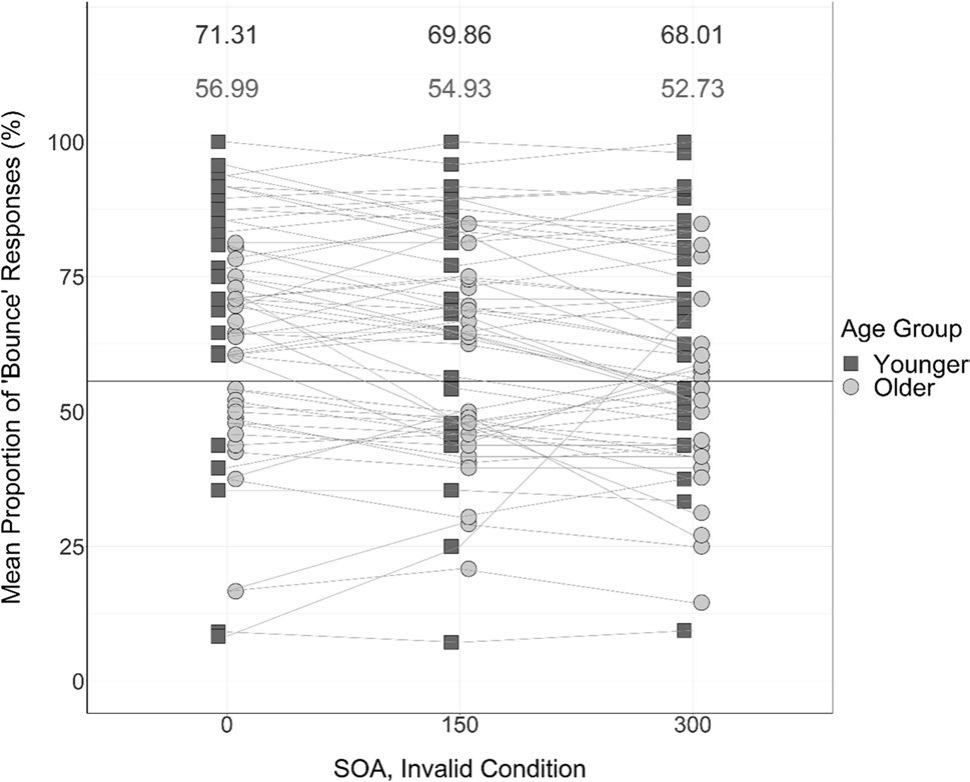
Mean proportion of “bounce” responses produced by each participant in each of the invalidly cued audiovisual conditions. Solid black horizontal reference line at 55.55% represents the mean proportion of “bounce” responses produced in the invalidly cued visual-only conditions. Black squares represent the data of younger adults; grey circles represent the data of older adults. Participant “bounce” responses are linked across conditions using lines. Numbers at the top of the figure display mean proportions of “bounce” responses in each condition—the means of younger adults are presented in the upper row in black; the means of older adults are presented in the lower row in grey

To illustrate the difference between the proportion of “bounce” responses in each of the audiovisual conditions compared with the visual-only control conditions, scatterplots were created with a horizontal reference line set at the mean proportion of “bounce” responses in the valid visual-only conditions (Fig. 8) and invalid visual-only conditions (Fig. 9), respectively.

#### Bounce/pass judgements: Unisensory conditions

It was first important to analyze the data from the 2 (age: younger vs older) × 2 (cue: valid vs invalid) mixed ANOVA that was conducted on the standardized “bounce” responses produced from the unisensory visual-only control conditions. In the visual-only ANOVA, there was no significant main effect of cue on the proportion of “bounce” responses, *F*(1, 58) = 0.00, *p* = 1.000, η_p_^2^ = 0.00, no significant main effect of age on the proportion of “bounce” responses, *F*(1, 58) = 2.31, *p* = .134, η_p_^2^ = 0.038, and no significant interaction between age and cue, *F*(1, 58) = 2.02, *p* = .161, η_p_^2^ = 0.034.

#### Bounce/pass judgements: Multisensory conditions

For the participants’ bounce/pass judgements in the audiovisual conditions, a 2 (age: younger vs older) × 2 (cue: valid vs invalid) × 3 (SOA: 0 ms vs 150 ms vs 300 ms) mixed ANOVA was conducted.

**Fig. 10:**
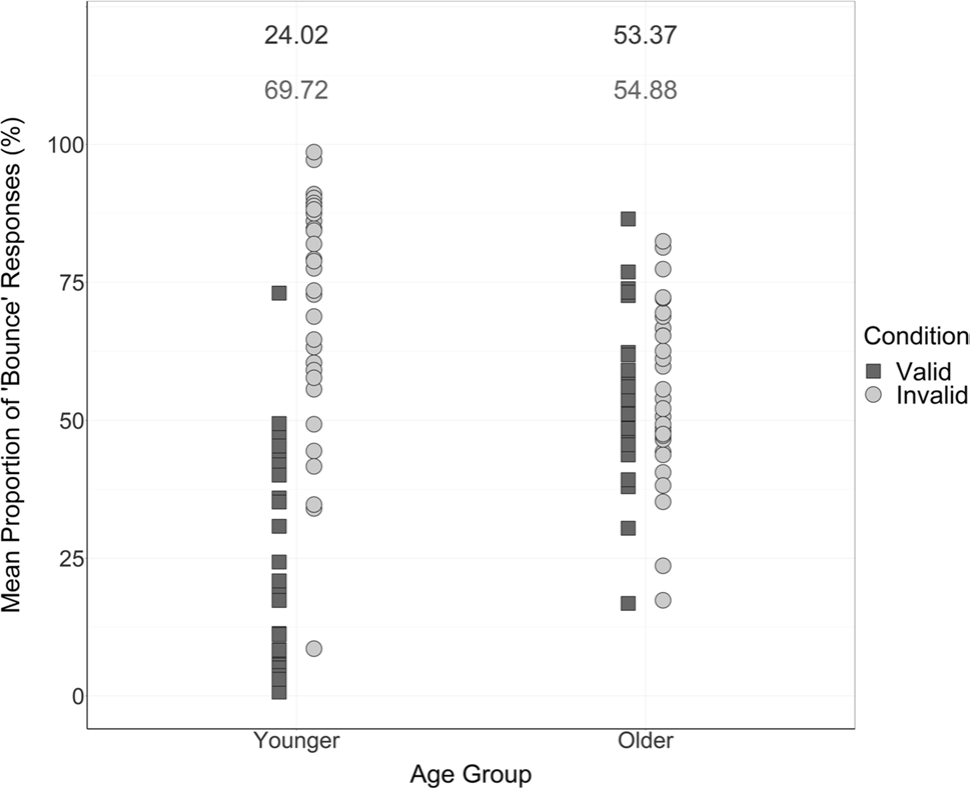
Mean proportion of “bounce” responses produced by each younger and older adult in validly cued and invalidly cued conditions. Black squares represent “bounce” judgements in valid conditions; grey circles represent “bounce” judgements in invalid conditions. Numbers at the top of the figure display mean proportions of “bounce” responses in each condition—the means produced in the valid condition are presented in the upper row in black; the means produced in the invalid condition are presented in the lower row in grey

To first assess whether there were differences in integration generally across age groups, the age variable in the 2 × 2 × 3 mixed ANOVA was examined. It was found that there was a significant main effect of age on the proportion of “bounce” responses, *F*(1, 58) = 5.29, *p* = .025, η_p_^2^ = 0.084. Overall, the proportion of “bounce” responses provided by older adults (*M* = 54.13%, *SE* = 2.23, 95% CI [49.66, 58.59]) was greater than the proportion of “bounce” responses provided by younger adults (*M* = 46.87%, *SE* = 2.23, 95% CI [42.41, 51.34]; mean difference = 7.26%, *SE* = 3.16), as displayed in Figs. 7 and 10. This suggests that older adults exhibited increased integration of the visual and auditory information compared with younger adults, which is an important finding as inefficient multisensory processing may be associated with increased risk of falls in older adults (Horak et al., 1989; Peiffer et al., 2007; Setti et al, 2011).

To investigate the effects of the attentional manipulation, it was important to assess the differences in validly cued vs invalidly cued conditions. The mixed ANOVA revealed a significant main effect of cue condition on the proportion of “bounce” responses, *F*(1, 58) = 43.40, *p* < .001, η_p_^2^ = 0.43. As displayed in Figs. 7 and 10, participants provided more “bounce” responses in the invalidly cued trials (*M* = 62.30%, *SE* = 2.45, 95% CI [57.39, 67.21]) compared with the validly cued trials (*M* = 38.70%, *SE* = 2.32, 95% CI [34.05, 43.34]; mean difference = 23.60%, *SE* = 3.58), in line with our hypothesis that the visual and auditory information is more likely to be perceived as synchronous and integrated in the invalidly cued condition (Donohue et al., 2015).

Mauchly’s test of sphericity indicated that the assumption of sphericity was violated for the SOA factor, χ^2^(2) = 36.72, *p* < .001. Greenhouse–Geisser adjusted *p*-values indicated that there was a significant main effect of SOA on “bounce” responses, *F*(1.36, 78.65) = 10.82, *p* < .001, η_p_^2^ = 0.16. Post hoc paired-samples *t* tests revealed that 0-ms trials produced a significantly greater proportion of “bounce” responses than did 150-ms trials, *t*(59) = 3.01, *p* = .004; mean difference = 2.16%, *SE* = 0.71; 300-ms trials, *t*(59) = 3.58, *p* = .001; mean difference = 4.39%, *SE* = 1.22; and visual-only trials, *t*(59) = 4.07, *p* < .001; mean difference = 8.27%, *SE* = 2.05. In addition, 150-ms trials produced a significantly greater proportion of “bounce” responses than 300-ms trials, *t*(59) = 2.77, *p* = .008; mean difference = 2.23%, *SE* = 0.82, and visual-only trials, *t*(59) = 3.35, *p* = .001; mean difference = 6.12%, *SE* = 1.84. Finally, 300-ms trials produced a greater proportion of “bounce” responses than visual-only trials, *t*(59) = 2.59, *p* = .012; mean difference = 3.89%, *SE* = 1.51. This is in line with previous research (Watanabe & Shimojo, 2001) suggesting that the temporal proximity of the visual and auditory information in the stream-bounce illusion influences whether they are integrated, with shorter SOAs producing more “bounce” responses. The descriptive statistics of these SOA comparisons are displayed in Table 3.

**Table 3:** Means and standard errors of the proportion of “bounce” responses provided at each level of the SOA condition (0 ms, 150 ms, 300 ms, visual-only)

	SOA			Visual-only
	0	150	300	
*M*, %	52.68	50.52	48.29	44.41
*SE*	1.85	1.65	1.66	1.85
95% CI	[49.14, 56.22]	[47.32, 53.73]	[45.03, 51.56]	[40.81, 48.00]

The interaction between age and cue was significant, *F*(1, 58) = 38.03, *p* < .001, η_p_^2^ = 0.40. This contrasts with the pattern of results found for the visual-only ANOVA, in which this significant interaction was not present. Our findings indicate that age and attention influence the multisensory integration of the auditory and visual information in this task. In line with our hypothesis, there were age-related differences in how the attentional manipulation affected multisensory integration and thus the proportion of “bounce” responses. As a result, it was necessary to analyze the pairwise comparisons of this interaction to investigate where these differences exist.

#### Bounce/pass judgements: Pairwise comparisons

To analyze pairwise comparisons within the age and cue interaction, the “bounce” responses in each audiovisual SOA condition were collapsed, so that a mean percentage of “bounce” responses provided by each participant could be calculated for validly cued and invalidly cued conditions. These percentages were then converted to standardized *z*-scores (see Statistical Analyses section).

##### Age pairwise comparisons

To assess differences between the proportion of “bounce” responses provided by younger adults and older adults in valid trials, and the differences between younger and older adults in invalid trials, two separate one-way ANOVAs were conducted (see Statistical Analyses section).

The first one-way ANOVA analyzed responses in the valid condition, and revealed that there were significant differences in the proportion of “bounce” responses between age groups, *F*(1, 58) = 40.03, *p* < .001. In the valid condition, a significantly greater proportion of “bounce” responses were produced by older adults (*M* = 53.37%, *SE* = 2.61, 95% CI [48.04, 58.70]) than younger adults (*M* = 24.02%, *SE* = 3.84, 95% CI [16.17, 31.87]).

In addition, the second one-way ANOVA analyzed responses in the invalid condition, and also indicated a significant difference between age groups, *F*(1, 58) = 9.15, *p* = .004. In the invalid condition, a significantly greater proportion of “bounce” responses were produced by younger adults (*M* = 69.72%%, *SE* = 3.97, 95% CI [61.61, 77.84]) than by older adults (*M* = 54.88%, *SE* = 2.89, 95% CI [48.97, 60.79]). These differences are displayed graphically in Fig. 10.

##### Cue pairwise comparisons

To assess differences in the proportion of “bounce” responses provided by younger adults in valid versus invalid trials, and by older adults in valid versus invalid trials, a repeated-measures ANOVA was conducted on the collapsed *z*-score data.

When examining the data of younger adults, the ANOVA revealed that there was a significant difference in the proportion of “bounce” responses in validly cued and invalidly cued trials, *F*(1, 29) = 47.76, *p* < .001, η_p_^2^ = 0.62. Overall, younger adults produced a significantly greater proportion of “bounce” responses in invalidly cued trials (*M* = 69.72%, *SE* = 3.97, 95% CI [61.61, 77.84]) compared with validly cued trials (*M* = 24.02%, *SE* = 3.84, 95% CI [16.17, 31.87]; mean difference = 45.71%, *SE* = 6.61).

However, when examining the data of older adults, it was revealed that there was no significant difference in the proportion of “bounce” responses in the validly cued and invalidly cued trials, *F*(1, 29) = 0.30, *p* = .589, η_p_^2^ = 0.01. Overall, older adults produced a similar proportion of “bounce” responses in the valid trials (*M* = 53.37%, *SE* = 2.61, 95% CI [48.04, 58.71]) as in the invalid trials (*M* = 54.88%, *SE* = 2.89, 95% CI [48.98, 60.79]; mean difference = 1.51%, *SE* = 2.76). Taken together, this suggests that, in line with our hypothesis, the multisensory integration of older adults was less affected by the attentional manipulation than younger adults. These differences are displayed in Fig. 10.

There was no significant interaction between cue and SOA, *F*(2, 116) = 0.42, *p* = .658, η_p_^2^ = 0.01, or between age and SOA, *F*(2, 116) = 1.21, *p* = .303, η_p_^2^ = 0.02. In addition, the three-way interaction between age, cue, and SOA was not significant, *F*(2, 116) = 1.06, *p* = .349, η_p_^2^ = 0.018. This means that conclusions cannot be made regarding how the width of the TBW, or the attentional modulation of the TBW, changes with healthy ageing.

## Discussion

The aim of this study was to investigate how the attentional modulation of the TBW changes as a function of ageing, replicating the paradigm of Donohue et al. (2015) to assess whether attentional cues can narrow the TBW in older adults in the same way that they were found to in younger adults. Upon analysis of the proportion of “bounce” responses produced in the unisensory visual-only conditions, as expected, there were no significant main effects of age or cue, and no significant interaction between age and cue. However, after analyzing the proportion of “bounce” responses produced in the multisensory audiovisual conditions, there were significant main effects of age and cue, and a significant interaction between age and cue. Arguably the most important finding of this study was that the attentional manipulation interacted with age in the multisensory conditions: spatial attention did not significantly influence the audiovisual integration of older adults, yet it did influence the integration of younger adults. This strongly suggests that older adults may have attentional deficits compared with younger adults, specifically associated with multisensory integration.

The crucial significant interaction between age and cue in the multisensory conditions was in line with our original hypothesis; younger adults produced a significant difference in the proportion of “bounce” responses between validly cued and invalidly cued conditions, and older adults produced a nonsignificant difference. If this finding indicates that older adults do have attentional deficits relative to younger adults (Gazzaley, 2013; Healey et al., 2008; Poliakoff et al., 2006), it suggests that older adults displayed increased difficulty in inhibiting task-irrelevant information when it co-occurs with task-relevant information, even when presented at the attended location (Fabiani, 2012).

It is important to note that much of the literature that argues the contrary—that attentional mechanisms remain unchanged between younger and older adulthood—is based upon selective and spatial attention experiments implementing very simple stimuli and tasks, such as identifying the colour of a circle, or identifying whether a visual flash or an auditory beep was presented first (de Dieuleveult et al., 2017; Hugenschmidt et al., 2009; Peiffer et al., 2007). The cued-spatial-attention version of the stream-bounce illusion utilized in the current study is comparatively much more difficult than this due to the higher cognitive demands and decisional elements of the task (Bedard & Barnett-Cowan, 2016); not only do participants need to process the attentional cue and the fast-moving visual stimuli, but if they integrate the auditory stimuli, participants must then also use their knowledge regarding how objects make a sound when they collide to inform their decision-making (Watanabe & Shimojo, 2001). It is therefore likely that the complex stimuli and complex task implemented in this experiment allowed for the detection of age-related deficits in attentional control, whereas previous research that found attentional mechanisms to be preserved in older adults may have observed somewhat of a ‘ceiling effect’, being unable to identify declines in attentional control due to the ease and simplicity of the tasks employed (Houx et al., 2002). Whilst it is a strength of the current study that the measures implemented were sensitive enough to uncover these important age-related attentional deficits in multisensory integration, this highlights how research investigating the mechanisms involved in multisensory integration, and how these change with age, appears to be highly task-dependent and stimuli-specific (Barutchu et al., 2019).

The significant main effect of cue in the multisensory conditions indicated that as hypothesized, a greater proportion of “bounce” responses was produced in invalidly cued conditions than in validly cued conditions. Previous literature surrounding attentional cueing (Posner, 1980; Posner & Driver, 1992) would suggest that one reason for this, specifically when analyzing the performance of younger adults, is that attending to the validly cued side inhibited the processing of task-irrelevant auditory information, reducing the likelihood of it being integrated with task-relevant visual information (Donohue et al., 2015; Mozolic et al., 2008; Talsma et al., 2007, 2010). This would explain the lower proportion of “bounce” responses provided by younger adults in the validly cued trials versus invalidly cued trials; their strong attentional control allowed them to focus on the “streaming” motion of the visual stimuli and decreased the influence of the distracting auditory information on the percept (Donohue et al., 2015; Kawabe & Miura, 2006).

A second, related reason for the significant main effect of cue could be that when the full “X” motion occurred on the unattended side of the screen, participants are likely to have missed the start of the movement and the crucial intersection (Donohue et al., 2015). This creates uncertainty about the visual stimuli, therefore perhaps participants relied more heavily upon the auditory information in the trial to make their pass/bounce judgements in these instances. This uncertainty, coupled with the knowledge that a sound usually occurs when two objects collide in everyday life (Watanabe & Shimojo, 2001), may have induced more “bounce” representations at the invalidly cued location, as attention was not present to enhance the full veridical movement of the visual stimuli (Donohue et al., 2015).

There was also a significant main effect of age, with older adults providing a significantly greater proportion of “bounce” responses overall compared with younger adults. This indicates that, in partial correspondence with our hypothesis, older adults integrated the visual and auditory information more than younger adults did. Previous research would suggest that this increased integration is due to the wider TBW of older adults providing a greater time span over which integration can occur (Brooks et al., 2018; Mozolic et al., 2012; Setti et al., 2011). However, we did not find a significant interaction between age and SOA, nor a significant interaction between age, SOA, and cue. Whilst a limitation of the current study is that the exact screen specifications of each participant could not be controlled because participants completed the experiment remotely, this is unlikely to be the sole explanation as to why a significant interaction was not found here.

One potential explanation as to why the SOA factor was not involved in any significant interactions could be due to the auditory element of the task eliciting demand characteristics (Nichols & Maner, 2008). That is, the mere presence of the sound in a trial could have induced a “bounce” response if participants believed that the experiment was simply measuring whether they detected the sound and related it to the perception of bouncing (McCambridge et al., 2012). If “bounce” responses were produced at either location simply due to the presence of the sound rather than the relative timing of the sound, attention was not specifically serving to “narrow” the TBW.

Importantly, studies that have successfully manipulated SOAs to find that older adults have a wider TBW (Laurienti et al., 2006; Mahoney et al., 2011; Peiffer et al., 2007; Setti et al., 2011) have used static stimuli such as flashes and beeps, whereas studies which have used dynamic visual stimuli (Roudaia et al., 2013; Stephen et al., 2010), like that in the stream-bounce illusion, did not detect such age-related changes in the width of the TBW. As such, the efficacy of systematically manipulating SOAs to index the width of the TBW may vary depending on whether the multisensory illusion uses static or dynamic stimuli (Roudaia et al., 2013). Previous research has postulated that dynamic stimuli may require increased processing within the visual modality before it is integrated with stimuli from other modalities (Stevenson & Wallace, 2013), which would result in a wider TBW. Perhaps longer SOAs are needed when implementing dynamic stimuli compared with static stimuli, to accurately index this wider TBW and detect differences between age groups.

In sum, the results of this study provide interesting directions for future research. Firstly, given that dynamic stimuli are more likely to index visual motion perception than static stimuli (Roudaia et al., 2013), future studies should focus on using moving visual stimuli like the stream-bounce illusion does, as this would result in more ecologically valid conclusions regarding how multisensory integration occurs in dynamic, everyday life environments. However, as suggested, perhaps longer SOAs should be used if dynamic stimuli are implemented, accounting for the increased time taken to process the stimuli within the modality before it is integrated with stimuli from other modalities (Stevenson & Wallace, 2013). This could increase the likelihood of detecting age-related changes in the width of the TBW.

Future, in-person research using neuroscientific techniques such as fMRI or TMS would allow for the investigation of the neurobiological origins of the bottom-up and top-down mechanisms involved in multisensory integration, and how they are affected by healthy ageing. Uncovering age-related changes in the magnitude and/or sequence of activation in different brain areas during multisensory processing is essential for understanding the relative contributions of mechanisms like the TBW and attentional control in the creation of an accurate and reliable percept of our environment. This knowledge is increasingly relevant as it could support the development of targeted programmes or therapies to strengthen the attentional control of older adults, sharpening their perception and reducing the risk of falls in our ageing population.

## Conclusion

To conclude, older adults in this experiment integrated more distracting, task-irrelevant information than younger adults. Crucially, however, the attentional manipulation within the task influenced audiovisual integration in older adults less than it influenced integration in younger adults, suggesting that older adults may have attentional deficits associated with multisensory integration. Manipulation of SOAs and assessing subsequent integration remains likely to be an effective way to index the width of the TBW; however, the stimulus specificity of the paradigms used must be considered. Future experiments employing dynamic stimuli could uncover more about how age-related changes in attentional control impact the temporal processing of multisensory stimuli, producing conclusions that are high in ecological validity. The findings of this would have significant practical applications in the development of clinical treatments to strengthen the attentional control of older adults, to enhance the temporal processing of task-relevant stimuli and inhibit the processing of distracting stimuli that should not be incorporated into the percept. Improving the multisensory perception of older adults in this way could greatly improve their ability to safely navigate through their environment and reduce their risk of falls.

## Data Availability

The datasets generated during and/or analyzed during the current study are available in the Lancaster University Pure repository (https://doi.org/10.17635/lancaster/researchdata/568).

## Code availability

The SPSS syntax used in the current study is available in the Lancaster University Pure repository (https://doi.org/10.17635/lancaster/researchdata/606).
